# Can the Carbon Emissions Trading System Improve the Green Total Factor Productivity of the Pilot Cities?—A Spatial Difference-in-Differences Econometric Analysis in China

**DOI:** 10.3390/ijerph19031209

**Published:** 2022-01-22

**Authors:** Dawei Huang, Gang Chen

**Affiliations:** 1 School of Management, Shenzhen Polytechnic, Shenzhen 518055, China; hdw@szpt.edu.cn; 2 School of Economics and Management, Harbin Institute of Technology (Shenzhen), Shenzhen 518055, China

**Keywords:** carbon emissions trading system, green total factor productivity, pilot cities, spatial difference-in-differences, difference-in difference-in-differences, energy efficiency, low-carbon innovation, industrial structure, spatial siphon effect, financial agglomeration, MRV capability

## Abstract

The carbon emission trading system (CETS) is an important market-oriented policy tool for the Chinese government to solve the problem of high emissions and achieve the growth of green total factor productivity (GTFP). This study makes up for the neglect of the spatial effect of CETS policy in previous studies and adopts the spatial difference-in-differences (DID) Durbin model (SDID-SDM) method of two-way fixed effects to scientifically identify the direct and spatial effects influencing the mechanisms and heterogeneity of CETS on urban GTFP based on the panel data of 281 cities in China from 2004 to 2017. It found that China’s CETS significantly improved the GTFP of pilot cities but produced a negative spatial siphon effect that restricted the growth of GTFP in surrounding cities. Benchmark results are robust under the placebo test, the propensity score matching SDID (PSM-SDID) test, and the difference-in difference-in-differences (DDD) test. The mechanism analysis shows that the CETS effect is mainly realized by improving energy efficiency, promoting low-carbon innovation, adjusting the industrial structure, and enhancing financial agglomeration. In addition, we find that policy effects are better in cities with high marketization, strong monitoring reporting and verification (MRV) capabilities, high coal endowment, and high financial endowment. Overall, China’s CETS policy achieves the goal of enhancing GTFP but needs to pay attention to the spatial siphon effect. In addition, our estimation strategy can serve as a scientific reference for similar studies in other developing countries.

## 1. Introduction

The climate problem is one of the most serious problems faced by mankind in the 21st century [1,2,3]. Existing studies have also confirmed that global temperature rise is closely related to greenhouse gas emissions [4], while carbon dioxide accounts for 84% of greenhouse gas emissions; it means that reducing carbon dioxide emissions is the key to solving the problem of climate deterioration [5,6]. Therefore, in response to the global climate crisis, countries around the world have taken the initiative to put “carbon peak” (many advanced economies have achieved “carbon peak”) and “carbon neutrality” on the agenda. As a major carbon emitter in recent years, China also pledged at the United Nations Climate Conference to strive to reach the peak before 2030 and to strive to achieve carbon neutrality by 2060 [7,8]. More and more studies have shown that the carbon emission trading system (CETS) plays an important role in reducing carbon dioxide emissions; for example, the United States Regional Greenhouse Emission Reduction Initiative (RGGI) [9,10], the European Union’s Carbon Emissions Trading System [11,12], South Korea’s 2015 “Greenhouse Gas Emission Allowance Allocation and Trading Act” [13] are important measures for advanced economies to achieve emission reduction targets, which are generally considered to be effective [14]. To cope with the severe greenhouse gas emission problem, in 2011, China officially approved the launch of carbon trading pilot projects in Beijing, Tianjin, Shanghai, Chongqing, Hubei, Guangdong, and Shenzhen, and in 2013–2014, the pilot areas successively launched carbon trading markets, accumulating experience for the construction of carbon markets in developing countries [15,16]. Domestic and foreign studies have shown that the carbon market is an effective policy tool to achieve specific emission reduction targets at a lower cost. Compared with traditional administrative management methods, it can not only transfer the responsibility of greenhouse gas emissions to enterprises but also provides corresponding economic incentives for carbon emission reduction and reduces the emission reduction costs of the whole society. It also drives green technological innovation and industrial investment and provides an effective tool for handling the relationship between economic development and carbon emission reduction [17,18]. Therefore, an in-depth understanding of the policy processing effects of the pilot carbon emissions trading policy is essential for the government to use market-based emission reduction tools to deal with the dual challenges of green sustainable development and high-quality economic growth.

In China, cities are the main spatial matrix of carbon emission governance. The provincial-level emission reduction political tasks issued by the central government will be decomposed to the city-level and implemented through the city-level government to specific emission reduction decisions of enterprises and institutions [19,20]. Therefore, city-level research will be more practical than provincial-level research.

Previous studies on the impact of CETS policies on GTFP have ignored the spatial effects of policies, resulting in a bias in the identification of policy effects [19]. There are two main sources of spatial effects of policies: one is the extensive internal circulation connection between cities. Although there are still some factor markets in China that have not been liberalized, the circulation of major commodities and factors between provinces and cities is smooth; that is, there are extensive internal circulation connections between cities. This internal circulation link will become a channel for the policy effect, acting in some regions to extend outside the pilot area, which will then generate the general equilibrium effect of the policy and form the spatial externality of the policy effect [21,22]. The second is the spatial redistribution of enterprises caused by enterprise location decisions under the pressure of CETS policy. Similar to the classic “pollution paradise hypothesis”, after the implementation of the CETS pilot policy, some carbon-emitting companies choose to relocate their factories to areas with lower environmental standards (such as non-pilot cities), which, in turn, produces a spatial externality of policy effects [19].

Existing studies have shown that CETS can achieve the policy effect of enhancing GTFP by improving energy efficiency, promoting low-carbon innovation, and adjusting industrial structure [23,24], but there is also the problem of ignoring the policy space effect. Under the pressure of CETS policy, carbon emission companies in pilot cities will take decisions such as carbon emission quota trading [25,26], emission reduction innovation, and location relocation to deal with the policy pressure [27]. At the macro level, these decisions drive industrial capital and economic factors to gradually shift from high-carbon industries to low-carbon and clean industries, which will lead to improvements in energy efficiency, low-carbon innovation, and a shift in industrial structure and then realize the promotion of urban GTFP at multiple scales, from the micro to the macro level [28,29,30]. At the same time, the differentiation of corporate emission reduction decisions will inevitably lead to the fact that some high-carbon companies cannot cover emission reduction costs through low-carbon innovation [31,32,33]. These manufacturers will choose to withdraw from the local market and relocate. On the one hand, it can improve the overall GTFP level of the original location, but on the other hand, the efficiency of the new location will be lowered due to their high-carbon decision-making, which will result in the spatial siphon effect of GTFP [34]. Furthermore, the intra-regional and inter-regional flow of industrial capital will have an impact on financial agglomeration and then affect the urban GTFP by affecting the efficiency of capital spatial allocation. This is another influence mechanism that is easily overlooked by existing research.

In addition, the problem of high carbon emissions brought about by China’s rapid economic growth is a typical problem that is occurring or will soon be faced by developing countries and some emerging market countries [19]. Therefore, our research can provide theoretical and practical experience for the construction of carbon markets in developing countries. Secondly, the CETS pilot areas approved by the Chinese government have different geographic locations and significant humane, economic, geographic, and spatial heterogeneous distribution characteristics. It is possible to comprehensively investigate the potential spatial heterogeneity of the CETS policy effects in regions with different economic development levels and different human and geographic characteristics [19]. In addition, as a typical market-oriented emission reduction policy tool, the policy effect of CETS will be affected by the level of marketization in the pilot area, the implementation of monitoring reporting and verification (MRV), and the structure of energy consumption [35]. Our research is of great help in understanding how CETS can improve GTFP under complex market economic conditions.

The marginal contributions of this study are mainly in three aspects: Firstly, we adopt the SDID-SDM model, with two-way fixed effects, based on prefecture-level city-level data rather than provincial-level data [36,37,38] to scientifically identify and estimate the direct impact and spatial lag impact of CETS on urban GTFP, which avoids identification bias due to missing spatial externalities of policy effects. Secondly, we use the panel data of 281 cities in mainland China to empirically examine the policy effects of China’s CETS, which provides additional quantitative evidence for the study of the policy performance of emission reduction tools such as carbon trading markets in emerging economies. It makes up for the lack of previous studies that have mainly focused on European and American markets [39] and ignored developing economies. Finally, based on the benchmark model, this research integrates spatial effects into the analysis of CETS’s mechanism of promoting GTFP and the analysis of heterogeneity of policy effects, which expands the spatial dimension of existing research content. In addition, we also discuss the CETS policy effect mechanism and the sources of heterogeneity from the perspective of financial agglomeration and financial endowment.

The rest of this paper is organized as follows: Section 2 discusses the policy background and development status of CETS. Part 3 presents the methodology and data. Section 4 presents the empirical results of the benchmark model and a series of robustness tests. The fifth part is the investigation of the CETS policy effect mechanism. Section 6 is an in-depth analysis of policy heterogeneity. The last part is the discussion and conclusions.

## 2. China’s Carbon Emission Trading System

**Figure 1:**
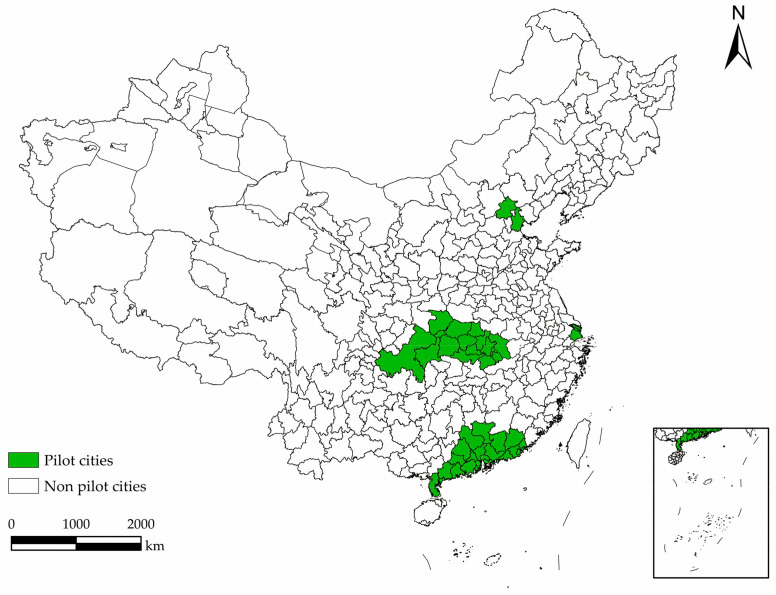
Distribution of CETS pilot areas in China.

The Chinese government has carried out a lot of policy exploration to deal with ecological and environmental issues in the context of high growth, especially carbon emissions [24]. In 2010, the State Council of China issued the “Decision on Accelerating the Cultivation and Development of Emerging Industries with Strategic Characters”, which formally proposed to “establish and improve a major pollutant and carbon emission trading system” as one of the tasks to deepen reforms in key areas. In 2011, China issued the “Outline of the Twelfth Five-Year Plan for National Economic and Social Development” and the “Comprehensive Work Plan for Energy Conservation and Emission Reduction in the Twelfth Five-Year Plan”. Both documents put forward the work objective of “carrying out pilot carbon emissions trading, establishing voluntary emission reduction mechanisms, and promoting the construction of a carbon emissions trading market”. In the same year, the National Development and Reform Commission of China issued the “Notice on Carrying out Pilot Carbon Emission Trading”, officially approving the seven provinces and cities of Beijing, Tianjin, Shanghai, Chongqing, Hubei, Guangdong, and Shenzhen to carry out carbon trading pilots (see Figure 1 for the distribution of pilot areas); in 2013–2014, pilot projects of carbon trading were successively launched.

In 2014, the National Development and Reform Commission of China issued the “Interim Measures for the Management of Carbon Emissions Trading”, which set a basic framework and mechanism for a nationwide carbon trading market, with “quota management”, “emissions trading”, “verification and allowance settlement”, “supervision and management” and many other dimensions.

Different from the CETS design based on the aggregate system generally adopted by developed economies, China’s CETS is mainly based on an intensity-based system design, which is essential to reduce the carbon emission intensity of economic activities.

As of July 2021, China’s carbon trading has gone through 7 periods of compliance. Judging from the statistical data officially released by China, CETS has achieved positive policy effects, as shown in Table 1 [40]. Statistics show that China’s CETS has exerted certain policy effects in terms of carbon dioxide emission reduction and economic benefits [24,41]. However, the statistics did not disclose the relevant content of the green development effect of the pilot policy; and as stated in the introduction, the current research on the effects of China’s CETS policy ignores the influence of regional spatial linkages on the role of policies [19].

Above all, the primary purpose of this study is to examine whether CETS can improve the GTFP of pilot cities under the condition of spatial effect by applying a scientific estimation model and to analyze the internal mechanism of the policy effect. In addition, as shown in Table 1, there is significant spatial heterogeneity in China’s carbon trading pilot statistics; that is, there are significant spatial differences among different carbon trading pilots in terms of industries covered, cumulative trading volume, cumulative trading volume, and carbon price. Therefore, the analysis of the heterogeneity of CETS policy effects must incorporate the spatial dimension into the analytical framework, which is another important research purpose of this study.

## 3. Methodology and Data

### 3.1. Methodology

We adopt a two-way fixed-effect SDID-SDM model to study the impact of CETS on GTFP. The combination of SDM and DID models can effectively identify the CETS policy effects with spatial dimensions [19]. The specific model settings are as follows:

(1) $$GTFP_{it}=\alpha_{0}+\rho W_{it}GTFP_{it}+\alpha_{1}DID_{it}+\alpha_{2}W_{it}DID_{it}+\sum_{j}\left(\beta_{j}control_{ijt}+\gamma_{j}W_{ijt}control_{ijt}\right)+city_{i}+year_{t}+\varepsilon_{it}$$

where *i* and *t* represent the city and year, respectively. *GTFP_it_* represents the urban green total factor productivity, and *W_it_* represents the spatial weight matrix for spatial lag effects, and *DID_it_* denotes the policy dummy variable of CETS. The control variables are represented by *controls_it_*. *city_i_*, *year_t_*, and *ε_it_* represent the city individual fixed effect, time fixed effect, and the random error term, respectively. According to the basic principles of the SDID-SDM model, this paper focuses on the estimated coefficients of the *DID_it_* and W*_it_* × *DID_it_* after controlling for other factors, which represent the direct and spatial lag impacts of CETS on urban GTFP.

Regarding the explained variables, essentially, GTFP is total factor productivity (TFP) considering environmental losses (pollution and carbon emissions, etc.); this is obtained by integrating energy input and environmentally undesired outputs on the basis of the original TFP. We use the Malmquist-Luenberger (ML) productivity index based on the slacks-based measure (SBM) directional distance function to measure the urban GTFP as an explained variable [42]. In the calculation process, the input variables selected in this paper include capital, labor, and energy consumption. Following the practice of most literature, this paper takes the capital stock as a proxy variable of capital investment, and specifically refers to the method of Liu et al. [43] to calculate the urban capital stock and make some adjustments. Labor input is approximately measured by the number of employees in urban units at the end of the period. Based on the availability principle, we measure the city’s energy consumption input with the city’s electricity consumption. Output variables include expected and undesired outputs. The expected output is expressed as the city’s real GDP after price deflation for the base period 2005. Undesirable outputs are CO_2_ emissions, industrial soot emissions, wastewater emissions, SO_2_ emissions, and PM2.5.

**Figure 2:**
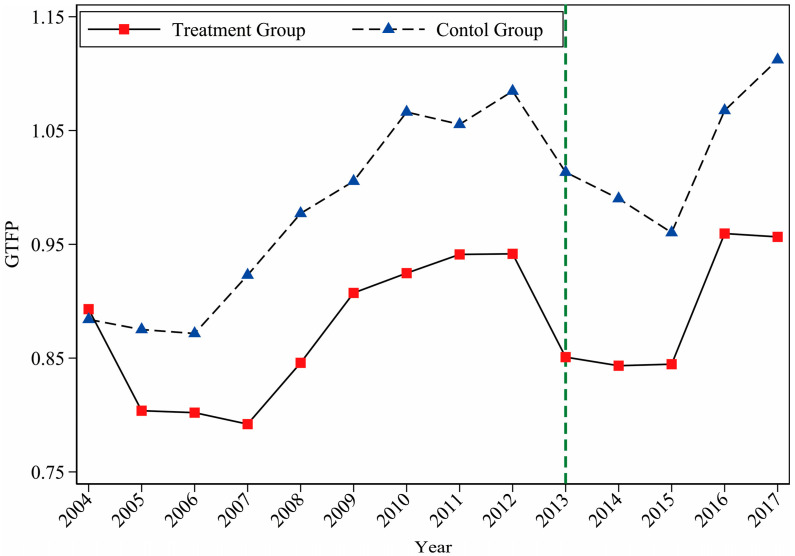
Changing trend of average GTFP between the treatment group and the control group.

Based on the calculated GTFP data, we plotted the changing trend of the average GTFP of the treatment group and the control group from 2004 to 2017, as shown in Figure 2. It is not difficult to see that before and after the pilot policy, the changing trend of the treatment group and the control group has changed. Before the implementation of CETS, the change trends of the two groups were the same, and the first four years after the implementation of the policy still maintained a certain degree of consistency, and there were significant differences in 2017. This preliminarily verifies that the treatment group and the control group meet the parallel trend assumption, and the subsequent empirical analysis will conduct a more scientific parallel trend test.

The key explanatory variable *DID_it_* is measured by *Treat_it_* × *Post_it_*, where *Treat_it_* represents the city grouping variable and *Post_it_* represents the time grouping variable. We let *Treat_it_* = 1 when city *i* is selected as the pilot area; otherwise, *Treat_it_* = 0, and when CETS is implemented in year *t*_0_, then ${\left.Post\right|}_{it}^{t\geq t_{0}}=1$.

Furthermore, to overcome the endogeneity problem caused by omitted variables when conducting the CETS policy effect assessment, that is, considering that the differences in GTFP between the treatment group and the control group before and after the CETS pilot policy implementation may have potential implications for policy evaluation [19,24], this paper controls some other variables that may affect urban GTFP, represented by *controls_it_*. Specifically, it includes economic development level (measured by per capita GDP), population size (measured by population density), energy consumption scale (measured by electricity consumption), and industrial structure (measured by industrial output value to GDP).

The regional characteristic factors at the province and city level and the time-invariant factors in a specific year will be controlled by a two-way fixed effect and clustering robust standard errors at the provincial level [38].

### 3.2. Samples and Data

As of the end of 2017, in addition to the seven pilot provinces and cities of Beijing, Tianjin, Shanghai, Chongqing, Hubei, Guangdong, and Shenzhen. Sichuan and Fujian provinces also established regional carbon markets at the end of 2016. In addition, in 2017, China’s National Development and Reform Commission issued the “National Carbon Emissions Trading Market Construction Plan (Power Generation Industry)”, which formally proposed the establishment of a national carbon emissions trading market. To avoid the interference of other provinces’ policy documents on the identification of regional pilot policy effects, we choose 2004–2017 as the sample period. Additionally, because of the heterogeneity of the CETS system design and regional endowments of the pilot provinces and cities, we use the fixed effect of the city and the fixed effect of the year to control the heterogeneous interference between the individual and the time level.

Considering the principle of data availability and the consistency of statistical coverage, when constructing dummy variables for groups, we excluded prefecture-level cities in the Tibet Autonomous Region, Hong Kong, Macau Special Administrative Region, and Taiwan, with serious data missing. Finally, 281 prefecture-level cities were selected as research samples, including 37 cities in the treatment group and 244 cities in the control group. The data required for empirical analysis mainly include two categories: input and output variables for measuring urban GTFP and city-level control variables for SDID-SDM analysis. The above variable data are all from the “China Urban Statistical Yearbook” and “China Statistical Yearbook” of the corresponding year. The urban CO_2_ emissions are calculated by superimposing and summing the carbon emissions of the counties under its jurisdiction.

## 4. Empirical Results

### 4.1. Parallel Trend Test

**Figure 3:**
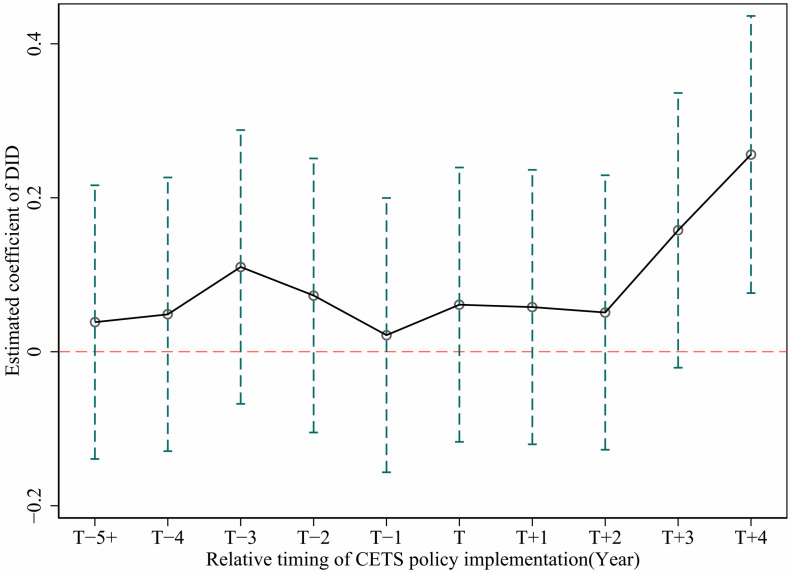
Parallel trend test.

The application of the DID method to evaluate CETS policy effects must meet the important “parallel trend assumption” [44,45], that is, the treatment group and the control group of the pilot policy should maintain a consistent change trend before being impacted by the policy, which is an important prerequisite for judging the effectiveness of SDID. Therefore, this paper adopts the event analysis method for parallel trend verification, as shown in Figure 3. It shows that none of the core explanatory variable coefficients before the CETS pilot policy passed the significance test, and after CETS was implemented, there was a significant difference between the two groups after a certain lag period. It means that the changing trend of GTFP in the treatment group and the control group is the same before the implementation of the policy; that is, it has passed the parallel trend test.

In addition, by observing the estimated coefficients of the core explanatory variables after the implementation of the policy, we find that the coefficients in the 1–3 years after the implementation of the CETS pilot policy were not significant, which means that within the first four years of the policy, CETS did not have an immediate policy effect on the promotion of urban GTFP.

### 4.2. Benchmark Results

As shown in Table 2, Columns (1) and (2) are the average treatment effect results estimated by the panel DID model and the SDID-SDM model. Column (3) is the result of the dynamic treatment effect of each year after the implementation of the pilot policy estimated by the SDID-SDM model. The results show that after controlling for the two-way fixed effect of the city’s individual effect and the year-time effect, without considering the spatial effect, CETS showed a negative inhibitory effect on the GTFP of the pilot cities. However, after considering the spatial effect, CETS significantly increased the GTFP of the pilot cities with a coefficient that was significant at the 10% confidence level; at the same time, it significantly suppressed the growth of GTFP in the surrounding areas, and the coefficients were all significant at the 1% confidence level. The difference between the two models is mainly due to the existence of spatial effects. The SDID-SDM model estimates that the coefficient of the DID term is positive and the coefficient of the spatial lag term is negative, indicating that the direct effect level CETS has positively improved the GTFP of the pilot cities. However, the overall effect is negative due to the larger negative spatial feedback effect, which explains the possible reasons for the different direction from the panel DID model results.

The dynamic effect analysis result of Column (3) shows that the CETS pilot policy showed a significant positive promotion effect in the fourth year after the implementation of the policy. The coefficient is significant at the 1% confidence level, and the promotion effect is increasing year by year. The impact of the policy on surrounding cities is manifested in the third year after the implementation of the policy, showing a negative spatial siphon effect, and the siphoning effect is increasing year by year.

The above benchmark regression results once again confirmed the significant improvement effect of CETS on the GTFP of the pilot cities, which is consistent with the existing research results [46]. The difference is that this research considers the spatial economic links between cities and adds the setting of policy spatial effects. The research shows that although CETS significantly increases the GTFP of the territorial cities, it also produces a negative spatial siphon effect on the surrounding cities, which is not conducive to the growth of GTFP in surrounding areas. We believe that this negative spatial siphon effect is mainly caused by the transfer of high-carbon companies from pilot cities to the surrounding areas under policy pressure. It is similar to the “pollution refuge” theory and can be understood as a “carbon emission refuge”.

Since there are spatial interaction terms of dependent variables in the benchmark SDID-SDM model, the benchmark model setting contains feedback effects between regions, so the model coefficients estimated by the benchmark regression need to be analyzed through effect decomposition and summation. Therefore, we report the effect decomposition of the core explanatory variables in the benchmark model, that is, the estimated coefficients of direct effects, indirect effects, and total effects. As shown in Table 3, the direct effect of the CETS policy effect coefficient is significantly positive at the 10% confidence level, and the indirect effect and the total effect are both significantly negative at the 1% confidence level.

### 4.3. Robustness Tests

#### 4.3.1. Placebo Test

**Figure 4:**
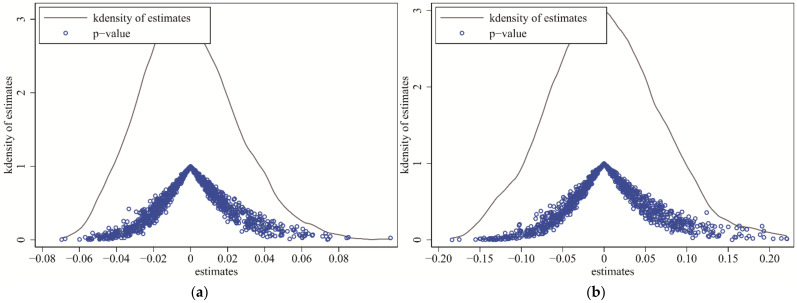
(**a**,**b**) are, respectively, the distribution diagrams of the coefficients of *DID* and *W* × *DID* on GTFP in the random sampling estimation results.

Based on the research method of Ren et al. [47], this paper conducts a placebo test by randomly assigning CETS pilot cities; that is, 37 cities are randomly selected as the treatment group for each sampling (the original number of the treatment group is 37), and the remaining 244 cities are used as the random control group. We performed 1000 independent replicates and estimations according to Equation (1). The distribution of the regression-estimated coefficients for independently repeated experiments is shown in Figure 4. It is not difficult to find that the distribution of estimated coefficients is concentrated around the zero point, and the benchmark estimated coefficients in Column (2) of Table 2 are on the edge of the distribution in the independently repeated experiments. It suggests that the enhanced effect of the CETS pilot policy on GTFP in pilot cities is unlikely to be driven by other exogenous factors, which means that it passes the placebo test.

#### 4.3.2. PSM-SDID Estimation

CETS has a significant promotion and negative spatial siphon effect on GTFP in pilot cities and the aforementioned parallel trend test has justified the application of the SDID method; however, considering that the non-random establishment of CETS pilot areas may lead to biased estimation results [48], we further adopt the propensity score matching SDID (PSM-SDID) method to avoid endogenous selection bias. We select urban economic development level, population size, energy consumption scale, and industrial structure as matching characteristic variables and then perform logistic regression and kernel matching to obtain matching samples and estimate them according to Formula (1). The results are shown in Column (1) of Table 4. It can be found that the coefficients of *DID_it_* and W*_it_* × *DID_it_* are significant at the 1% level, which indicates that the benchmark regression estimation results in Table 2 are robust.

#### 4.3.3. The Difference-in Difference-in-Differences (DDD)

A possible problem with the SDID estimation strategy described above is that there may be other policies in addition to the CETS pilot policy that have inconsistent effects on GTFP in pilot and non-pilot regions. The interference of these policies can confound the estimates of the baseline model. For example, the ETS pilot policy has been implemented in 11 provinces (cities), including Tianjin, Hebei, Shanxi, Inner Mongolia, Jiangsu, Zhejiang, Henan, Hubei, Hunan, Chongqing, and Shaanxi, since 2007. We control the possible confusing effects of ETS by constructing dummy variables for carbon emissions trading pilots [38] and then use the difference-in difference-in-differences (DDD) method to overcome this problem. Column (2) of Table 4 reports the average treatment effect estimated by DDD, and the results are consistent with the baseline estimates, indicating that the CETS pilot policy effects estimated by the baseline model are reliable.

## 5. Mechanism Analysis

### 5.1. Re-Examination of Traditional Mechanisms

As discussed in the introduction, previous studies have shown that CETS will influence changes in urban-level GTFP through channels such as energy efficiency, low-carbon technological innovation, and industrial structure. However, these studies often overlook the important role that spatial effects may play in the influence mechanism. Therefore, we will empirically re-examine these potential impact mechanisms.

#### 5.1.1. Energy Efficiency Effect

Previous studies focused on analyzing the impact of companies choosing to improve energy efficiency on GTFP in territorial cities [49], and they ignore the economic efficiency brought about by local energy efficiency improvements, which will attract companies with higher energy efficiency in the surrounding areas to migrate to the area. At the same time, there are still some companies in this area that cannot cover costs even if they improve energy efficiency. These companies often choose to move to the surrounding areas. The dual effects of high input and low output jointly produce a positive promotion effect on the territorial cities and a spatial siphon effect of negative adsorption on the surrounding areas. We approximate urban energy efficiency as the ratio of urban industrial electricity consumption to GDP, and the lower the value, the higher the energy efficiency of the city. Therefore, the estimation result of the prediction model is exactly opposite to the estimation result of the benchmark regression model. We apply the following model to test the energy efficiency effect:

(2) $$\begin{array}{l} GTFP_{it}=\alpha_{0}+\rho W_{it}GTFP_{it}+\sum_{j}\left(\beta_{j}control_{ijt}+\gamma_{j}W_{ijt}control_{ijt}\right)+city_{i}+year_{t}+\varepsilon_{it}\\ +(\alpha_{1}DID_{it}+\alpha_{2}ee_{it}+\alpha_{3}DID_{it}\times ee_{it})+(\delta_{1}W_{it}DID_{it}+\delta_{2}W_{it}ee_{it}+\delta_{3}W_{it}(DID_{it}\times ee_{it})) \end{array}$$

Column (1) in Table 5 reports the estimated results of the energy efficiency effect identified based on Formula (2). The results in the table show that the coefficients of the core explanatory variables are significant at the 1% level, and, consistent with the previous assumption, CETS can improve the energy efficiency of the territorial cities to achieve a positive effect on the GTFP of the territorial cities and a negative spatial siphon effect on the surrounding areas.

#### 5.1.2. Low-Carbon Innovation Effect

Similar to energy efficiency, we believe that CETS can achieve a positive, improving effect on the territorial city and a negative spatial siphon effect on the surrounding area by promoting the low-carbon innovation of the territorial city [50]. We use the ratio of the number of low-carbon patents to the total number of patent grants to measure the level of city-level low-carbon innovation and use the following model to test the low-carbon innovation effect:

(3) $$\begin{array}{l} GTFP_{it}=\alpha_{0}+\rho W_{it}GTFP_{it}+\sum_{j}\left(\beta_{j}control_{ijt}+\gamma_{j}W_{ijt}control_{ijt}\right)+city_{i}+year_{t}+\varepsilon_{it}\\ +(\alpha_{1}DID_{it}+\alpha_{2}lci_{it}+\alpha_{3}DID_{it}\times lci_{it})+(\delta_{1}W_{it}DID_{it}+\delta_{2}W_{it}lci_{it}+\delta_{3}W_{it}(DID_{it}\times lci_{it})) \end{array}$$

Column (2) of Table 5 reports the estimation results of the low-carbon innovation effect identified based on Formula (3). The data in the table show that the coefficients of the core explanatory variables are significant at the 5% and 10% levels, respectively, and are consistent with the previous assumptions. CETS can realize the positive promotion effect on the GTFP of the local city and the negative spatial siphon effect on the surrounding areas by promoting the low-carbon innovation of the local city.

#### 5.1.3. Industry Structure Effect

When faced with CETS policy pressure, individual companies in pilot cities will often choose to purchase allowances for carbon emission, improve energy efficiency, low-carbon technology innovation, and migration based on their emission reduction costs and benefits. However, no matter which option, there are more enterprise groups in the entire market environment. For example, if a company buys quotas, it inevitably means that a company sells quotas. This will form a structural transformation process; that is, under the pressure of CETS policy, companies will gradually withdraw from high-carbon industries and transition to green and low-carbon industries. This change in industrial structure will have a positive impact on urban GTFP. Similarly, previous studies have focused on analyzing the impact of changes in the industrial structure of pilot cities on GTFP in territorial cities while ignoring the process of entry and exit of enterprises between regions. The dual effects of high industrial allocation efficiency in and low out have jointly produced a positive promotion effect on the territorial cities and a spatial siphon effect of negative absorption on the surrounding areas. We use the proportion of the secondary industry to measure the city-level industrial structure. Theoretically, the estimation result is exactly opposite to the estimation result of the benchmark regression model. We adopt the following model to test the industry structure effect:

(4) $$\begin{array}{l} GTFP_{it}=\alpha_{0}+\rho W_{it}GTFP_{it}+\sum_{j}\left(\beta_{j}control_{ijt}+\gamma_{j}W_{ijt}control_{ijt}\right)+city_{i}+year_{t}+\varepsilon_{it}\\ +(\alpha_{1}DID_{it}+\alpha_{2}str_{it}+\alpha_{3}DID_{it}\times str_{it})+(\delta_{1}W_{it}DID_{it}+\delta_{2}W_{it}str_{it}+\delta_{3}W_{it}(DID_{it}\times str_{it}) \end{array}$$

Column (3) of Table 5 reports the estimated results of the industry structure effect identified based on Formula (4). The data in the table show that the coefficient of the core explanatory variable is significant at the 1% level. Consistent with the previous assumptions, CETS can improve urban GTFP by reducing the proportion of secondary industry in pilot cities; by increasing the proportion of secondary industries in surrounding cities, a negative spatial siphon effect is generated.

### 5.2. Financial Agglomeration Effect

The traditional mechanism analysis mainly focuses on the efficiency and structural level of the influence mechanism while ignoring that the effect of CETS policy may be realized through the accumulation of financial capital. When analyzing how CETS affects urban GTFP through industrial structure, we believe that CETS will guide the structural transfer of industrial capital. Specifically, when companies adopt green and low-carbon technological innovations to respond to the pressure to reduce emissions, they will attract green and low-carbon capital to settle in the pilot city. When companies face the pressure to reduce emissions and choose to flee the pilot cities, some high-carbon capital will be withdrawn. This kind of capital entry and exit is related to corporate emission reduction decisions. It means that CETS may have an impact on the spatial distribution of financial capital, which, in turn, will affect the spatial allocation efficiency of financial elements and change the city’s GTFP. We use the location entropy of the financial industry to measure the level of financial agglomeration at the city level [51]. We apply the following model to test the financial agglomeration effect:

(5) $$\begin{array}{l} GTFP_{it}=\alpha_{0}+\rho W_{it}GTFP_{it}+\sum_{j}\left(\beta_{j}control_{ijt}+\gamma_{j}W_{ijt}control_{ijt}\right)+city_{i}+year_{t}+\varepsilon_{it}\\ +(\alpha_{1}DID_{it}+\alpha_{2}fa_{it}+\alpha_{3}DID_{it}\times fa_{it})+(\delta_{1}W_{it}DID_{it}+\delta_{2}W_{it}fa_{it}+\delta_{3}W_{it}(DID_{it}\times fa_{it})) \end{array}$$

Column (4) of Table 5 gives the estimated results of the financial agglomeration angle. The results show that CETS can achieve the effect of improving the city’s GTFP by increasing the financial agglomeration of pilot cities, and it produces a negative spatial siphon effect on the financial agglomeration of surrounding cities, and then produces a negative policy effect that inhibits GTFP in surrounding areas.

## 6. Heterogeneity Analysis

Theoretically, the market-based trading of allowances can equalize the marginal abatement costs of various emitters and control emissions at the lowest cost. The key to the policy effect of CETS is that the carbon price information can truly and accurately reflect carbon market emissions information, which depends on the implementation of monitoring, reporting, and verification (MRV). Market-oriented policy tools and MRV are both depend on a relatively free-market economic environment. Therefore, the marketization level and MRV capacity of carbon trading market pilot cities are important factors that lead to the difference in the policy effect of CETS on GTFP. In addition, there are significant differences in energy consumption endowments between resource-based cities rich in coal and oil and non-resource cities. This endowment difference makes different cities have different policy effects when facing CETS policy pressure [52]. Moreover, differences in financial endowments not only affect the investment and financing process of low-carbon industries but also affect the allocation structure and efficiency of financial capital among industries. Therefore, we believe that urban financial endowment is also an important source of heterogeneity affecting policy effects.

We measure the above four heterogeneity factors by the following methods. Firstly, we divide the provincial marketization data as grouping dummy variables named *Mar* (cities in the high-level group mean *Mar* = 1, and cities in the low group mean *Mar* = 0) based on the median line to study the heterogeneity of policy effects that may be brought about by the level of marketization [19,36,53]. Secondly, we learn from Wang S S et al. (2021) [19] and use the ratio of provincial environmental administrative penalty cases to total energy consumption to measure the level of regional environmental enforcement, which is used to approximate urban MRV capability. We group the ratio by median named *Mrv* (cities in high group mean *Mrv* = 1, and cities in low group mean *Mrv* = 0). Thirdly, we calculate the proportion of provincial coal consumption in energy consumption (group proportional data by median named *ECE* (cities in the high group mean *ECE* = 1, and cities in the low group mean *ECE* = 0)) to study the policy heterogeneity that energy consumption structure may bring. Finally, we use the proportion of the total balance of deposits and loans of financial institutions in the city at the end of the year to the regional GDP to measure the level of urban financial endowment (group proportional data by median named *Fin* (cities in the high group mean *Fin* = 1, and cities in the low group mean *Fin* = 0)). For all these grouping processes, we mainly use provincial data from the year before the pilot policy as the basis for grouping to avoid possible selection bias.

As shown in Table 6 Columns (1)–(4), we estimated the coefficients of the core explanatory variables under the four heterogeneity groups, namely, marketization level, MRV capacity, energy consumption structure, and financial endowment. It is not difficult to find that the coefficients of the DID term and W × DID term, estimated by the four heterogeneous sources, are all in the same direction as the baseline regression estimation result. It shows that the policy effect of CETS’s promotion of GTFP in pilot cities shows significant heterogeneous characteristics under different marketization levels, environmental law enforcement efforts, and energy consumption endowments. Moreover, the policy effects perform better in regions with a high degree of marketization, strong MRV capabilities, a high proportion of coal consumption, and better financial endowments.

## 7. Discussion and Conclusions

### 7.1. Discussion

This paper regards the CETS pilot policy as a quasi-natural experiment. Based on panel data of 281 cities in China from 2004 to 2017, we adopt the SDID-SDM model with two-way fixed effects to investigate the impact of China’s CETS on urban GTFP. After controlling the influence of individual fixed effects, time fixed effects, and controlled variables, CETS has significantly improved GTFP in pilot cities, which is consistent with the conclusions of existing related research [46]. However, the ignorance of spatial effects in these studies makes the identification of policy effects biased. We applied the SDID-SDM model to effectively identify the spatial effects of CETS policies.

Subsequently, we introduced spatial effects in the empirical test of the three influencing mechanisms in traditional research, namely, energy efficiency, low-carbon innovation, and industrial structure. While verifying the conclusions of previous studies, the performance of the influence mechanism at the level of spatial effects was investigated. The decisions made by the pilot city’s carbon emission companies in the face of CETS policy pressure not only affect the city’s internal economic micro and macro performance, but it will also affect GTFP in surrounding areas through enterprises moving in and out, commodity trade links, and the circulation of resource elements. It is an important source of significant spatial effects at the level of the impact mechanism. In addition, previous studies have overlooked the process of spatial allocation of financial capital brought about by changes in economic structure, which is also an important channel of how CETS affects urban GTFP. We introduce financial agglomeration to effectively identify the influence mechanism of this process. Follow-up research can try to start from the perspective of enterprise entry and exit, analyze the impact of CETS on the location choice of carbon emission companies, and investigate in-depth whether the impact will promote the improvement of GTFP in the region.

To thoroughly investigate the source of the heterogeneity of policy effects, we combine the market-oriented characteristics of the CETS policy, the policy’s high dependence on the government’s MRV capabilities, and the energy consumption structure of the regions where the policy is implemented and analyze the heterogeneity of policy effects. Similar to previous research conclusions, the level of marketization and the government’s MRV capability are effective guarantees for pilot cities to exert the effects of CETS policies. The dependence of cities on high-emission energy sources such as coal is more conducive to CETS’s policy effectiveness. This result, on one hand, confirms that China’s market reform has achieved certain results and also refutes the doubts of some foreign scholars [38]. It also shows that the spatial heterogeneity of China’s internal marketization level is a key factor affecting the effects of CETS policy. On the other hand, it also affirms the growth of the Chinese government in MRV capabilities; at the same time, it also verifies the key influence of MRV capability on CETS’s policy effect. In addition, we also investigated the impact of urban financial endowment on CETS’s policy effects. The results show that financial endowment is also an important source of heterogeneity. Subsequent research can consider introducing variables related to the green finance index and in-depth analysis of the effect of green finance on CETS’s policy effectiveness and its internal mechanism.

### 7.2. Conclusions

In general, our research provides spatial-level test evidence for the role, mechanism, and heterogeneity of CETS’s impact on urban GTFP. It makes up for the negligence of previous related studies on the policy spatial effect. In addition, the identification method of this study can provide a scientific reference for other high-emission developing countries to carry out similar studies.

Although this research provides evidence of additional econometric analysis for the government in carbon trading market management decision-making and green development research, there are still some directions for improvement. Firstly, our research object only focused on 281 cities in mainland China and we did not examine the policy effects, mechanisms, and heterogeneity of carbon trading market policies in other emerging market economies or developing countries from a spatial dimension, which means there is a lack of broader empirical arguments. Secondly, although we have introduced financial agglomeration and financial endowments into the analysis framework, it did not launch a more in-depth analysis. In addition, we have not compared the pilot policy with the national carbon market that has been in operation. These will be feasible directions for follow-up research.

## Figures and Tables

**Table 1:** Current status of the pilot carbon emission trading market.

Pilot Area	Trading Varieties	Covered Industries	Cumulative Trading Volume	Cumulative Turnover (100 Million Yuan)	Current [Lowest, Highest] Price (Yuan/Ton)
Beijing	CO_2_	Heating power, electric power, cement, petrochemical	0.15	9.04	50.30 [24.00, 102.96]
Tianjin	CO_2_	Steel, fossil, electric power, thermal power, petrochemical, oil and gas extraction	0.19	4.08	29.86 [7.00, 62.38]
Shanghai	CO_2_	Steel, petrochemical, chemical, electric power, non-ferrous metals, building materials, textiles, paper, rubber, chemical fiber, aviation, airports, ports, railways, commerce	0.17	5.18	39.00 [4.21, 49.93]
Chongqing	CO_2_, CH_4_, etc.	Electrolytic aluminum, titanium alloy, calcium carbide, caustic soda, cement, steel	0.09	0.42	32.67 [1.00, 44.86]
Shenzhen	CO_2_	Electricity, taxation, construction, manufacturing, transportation	0.49	11.80	13.34 [3.12, 122.97]
Guangdong	CO_2_	Electricity, cement, steel, petrochemical, ceramics, textile, paper, non-ferrous metals	1.68	33.02	43.44 [1.27, 77.00]
Hubei	CO_2_	Steel, electricity, cement, chemicals, petrochemicals, automobile manufacturing, non-ferrous metals, glass building materials, papermaking, chemical fiber, pharmaceuticals, food and beverages	0.75	17.02	31.81 [9.38, 54.64]

**Table 2:** Baseline regression results.

Model	Panel-DID	SDID-SDM	
Variables	(1)	(2)	(3)
DID	−1.235 ***	0.083 *	
	(−9.85)	(1.80)	
W × DID		−0.172 ***	
		(−3.04)	
Treat × year13			0.028
			(0.33)
Treat × year14			0.025
			(0.29)
Treat × year15			0.018
			(0.21)
Treat × year16			0.125
			(1.46)
Treat × year17			0.223 ***
			(2.58)
W × treat × year13			−0.096
			(−0.90)
W × treat × year14			−0.069
			(−0.65)
W × treat × year15			−0.027
			(−0.26)
W × treat × year16			−0.189 *
			(−1.78)
W × treat × year17			−0.489 ***
			(−4.58)
Control	Y	Y	Y
Year-FE	Y	Y	Y
City-FE	Y	Y	Y
Obs.	3934	3934	3934
R^2^	0.351	0.112	0.111

Note: DID is short for difference-in-differences; SDID-SDM is short for spatial difference-in-differences Durbin model; FE is short for fixed effect. The parentheses are the *t*-values. *** and * represent significant levels at 1% and 10%, respectively.

**Table 3:** Decomposition of the spatial effect of CETS: direct effect, indirect effect, and total effect.

Variable	Direct Effect	Indirect Effect	Total Effect
DID	0.081 **	−0.179 ***	−0.098 ***
	(1.75)	(−3.13)	(−3.02)

Note: DID is short for difference-in-differences. The parentheses are the *t*-values. *** and ** represent significant levels at 1%, and 5%, respectively.

**Table 4:** Estimation results in PSM-SDID and effect with ETS.

Model	PSM-SDID	ETS
Variables	(1)	(2)
DID	0.140 ***	
	(2.91)	
W × DID	−0.232 ***	
	(−3.99)	
DDD		0.145 ***
		(3.03)
W × DDD		−0.233 ***
		(−4.02)
Control	Y	Y
Year-FE	Y	Y
City-FE	Y	Y
Obs.	3934	3934
R^2^	0.123	0.271

Note: DID is short for difference-in-differences; PSM-SDID is short for propensity score matching spatial difference-in-differences; ETS is short for emissions trading system; DDD is short for difference-in difference-in-differences; FE is short for fixed effect. The parentheses are the *t*-values. *** represent significant levels at 1%, respectively.

**Table 5:** Results of the impact mechanism analysis.

Model	Energy Efficiency	Low Carbon Innovation	Industry Structure	Financial Agglomeration
Variables	(1)	(2)	(3)	(4)
DID × ee	−0.177 ***			
	(−3.69)			
W × DID × ee	0.526 ***			
	(6.20)			
DID × lci		0.886 **		
		(2.24)		
W × DID × lci		−8.628 *		
		(−1.74)		
DID × str			−1.202 ***	
			(−4.19)	
W × DID × str			2.093 ***	
			(3.69)	
DID × fa				0.046 ***
				(3.21)
W × DID × fa				−0.092 ***
				(−3.50)
Control	Y	Y	Y	Y
Year-FE	Y	Y	Y	Y
City-FE	Y	Y	Y	Y
Obs.	3934	3934	3934	3372
R^2^	0.177	0.117	0.118	0.119

Note: DID is short for difference-in-differences; FE is short for fixed effect. The parentheses are the *t*-values. ***, **, and * represent significant levels at 1%, 5%, and 10%, respectively.

**Table 6:** Heterogeneity analysis results.

Model	Marketization Level	MRV Capability	Energy Consumption Endowment	Financial Endowment
Variables	(1)	(2)	(3)	(4)
DID × Mar	0.956 ***			
	(5.25)			
W × DID × Mar	−0.289 **			
	(−1.96)			
DID × Mrv		0.227 ***		
		(2.62)		
W × DID × Mrv		−0.402 ***		
		(−3.64)		
DID × ECE			0.155 *	
			(1.81)	
W × DID × ECE			−0.386 ***	
			(−3.89)	
DID × Fin				0.049 ***
				(3.41)
W × DID × Fin				−0.092 ***
				(−3.50)
Control	Y	Y	Y	Y
Year-FE	Y	Y	Y	Y
City-FE	Y	Y	Y	Y
Obs.	3934	3934	3934	3372
R^2^	0.110	0.117	0.121	0.098

Note: DID is short for difference-in-differences; MRV is short for monitoring reporting and verification; Mar is short for marketization level; ECE is short for energy consumption endowment; Fin is short for financial endowment; FE is short for fixed effect. The parentheses are the *t*-values. ***, **, and * represent significant levels at 1%, 5%, and 10%, respectively.

## Data Availability

The data presented in this study are available on request from the corresponding author. The data are not publicly available due to privacy.
